# ProKaSaRe Study Protocol: A Prospective Multicenter Study of Pulmonary Rehabilitation of Patients With Sarcoidosis

**DOI:** 10.2196/resprot.4948

**Published:** 2015-12-04

**Authors:** Heidrun Lingner, Anika Großhennig, Kathrin Flunkert, Heike Buhr-Schinner, Rolf Heitmann, Ulrich Tönnesmann, Jochen van der Meyden, Konrad Schultz

**Affiliations:** ^1^ Centre for Public Health and Healthcare Hannover Medical School Hannover Germany; ^2^ Institute of Biostatistics Hannover Medical School Hannover Germany; ^3^ Ostseeklinik Schönberg Holm Centre for Rehabilitation, Pneumology, Cardiology and Orthopedics Schönberg-Holm Germany; ^4^ Centre for Rehabilitation, Pneumology and Cardiology MediClin Albert Schweitzer Klinik Königsfeld Germany; ^5^ Center for Rehabilitation, Pneumology and Oncology Wehrawald Clinic Todtmoos Germany; ^6^ Bad Reichenhall Clinic Centre for Rehabilitation, Pneumology and Orthopedics Bad Reichenhall Germany

**Keywords:** sarcoidosis, rehabilitation, quality of life, fatigue

## Abstract

**Background:**

Available data assessing the efficacy of pulmonary rehabilitation for patients with chronic sarcoidosis are scant; for Germany, there are none at all.

**Objective:**

To gain information about the benefit of in-house pulmonary rehabilitation for patients with chronic sarcoidosis and for the health care system, we intend to collect data in a prospective multicenter “real-life” cohort trial.

**Methods:**

ProKaSaRe (Prospektive Katamnesestudie Sarkoidose in der pneumologischen Rehabilitation) [Prospective Catamnesis Study of Sarcoidosis in Pulmonary Rehabilitation] will assess a multimodal 3-week inpatient pulmonary rehabilitation program for adult patients with chronic sarcoidosis over a 1-year follow-up time. Defined specific clinical measurements and tests will be performed at the beginning and the end of the rehabilitation. In addition, questionnaires concerning health-related quality of life and the patients’ symptoms will be provided to all patients. Inclusion criteria will be referral to one of the 6 participating pulmonary rehabilitation clinics in Germany for sarcoidosis and age between 18 and 80 years. Patients will only be excluded for a lack of German language skills or the inability to understand and complete the study questionnaires. To rule out seasonal influences, the recruitment will take place over a period of 1 year. In total, at least 121 patients are planned to be included. A descriptive statistical analysis of the data will be performed, including multivariate analyses. The primary outcomes are specific health-related quality of life (St George’s Respiratory Questionnaire) and exercise capacity (6-minute walk test). The secondary outcomes are several routine lung function and laboratory parameters, dyspnea scores and blood gas analysis at rest and during exercise, changes in fatigue, psychological burden, and generic health-related quality of life (36-item Short Form Health Survey).

**Results:**

Funding was obtained on October 12, 2010; enrollment began on January 15, 2011 and was completed by January 14, 2012. Results are anticipated late summer 2015.

**Conclusions:**

Due to the large number of participants, we expect to obtain representative findings concerning the effectiveness of pulmonary rehabilitation for patients with sarcoidosis and to provide a dataset of assessed objective and subjective short- and long-term changes due to pulmonary rehabilitation. The results should form the basis for the planning of a randomized controlled trial.

**Trial Registration:**

German Clinical Trials Register: DRKS00000560; https://drks-neu.uniklinik-freiburg.de/ drks_web/navigate.do?navigationId=trial.HTML&TRIAL_ID=DRKS00000560 (Archived by WebCite® at http://www.webcitation.org/6dKb5X87R)

## Introduction

Sarcoidosis is a granulomatous systemic disease of still unknown etiology [1] that seemingly is caused by a combination of genetic and environmental factors [2]. The lungs and thoracic lymph nodes are impaired in approximately 90% of patients [3,4]. Because of the systemic nature of the disease, any organ (eg, eyes, heart, skin, kidneys, liver, and the central nervous system) can be affected [5]. Due to its various manifestations, sarcoidosis shows very heterogeneous clinical pictures [6-8].

The incidence and prevalence of sarcoidosis have not been precisely compiled for Germany. Estimates using data from the Bonn Sarcoidosis collective give a prevalence of approximately 44 per 100,000 inhabitants [9]. Sarcoidosis may occur at any age, although the age distribution peaks between 20 and 40 years; women are more affected than men [9]. Therapeutic strategies aim to reduce the progressing sarcoidosis symptoms. Spontaneous remissions occur in a high percentage of cases. Nevertheless, more than 20% of sarcoidosis patients will develop a chronic or recurrent form of the disease; up to 10% suffer serious complications such as lung fibrosis or cardiac symptoms. Unfortunately, up to 5% of all cases end fatally [9].

Depending on the affected organs, sarcoidosis may cause functional limitations and reduce quality of life. It is mostly accompanied by a pronounced fatigue [9-11] and myasthenia [12]. Systemic corticosteroids remain the treatment of choice when organ function is threatened or progressively impaired [13,14]. The muscle atrophy in sarcoidosis patients is the main reason why, in addition to medication, a rehabilitation program with particular emphasis on training [15] in a multidisciplinary treatment and management setting is strongly recommended for sarcoidosis patients [11,16-19]. In contrast to rehabilitation for patients with chronic obstructive pulmonary disease (COPD), the available data assessing the efficacy of pulmonary rehabilitation for patients with sarcoidosis are sparse; for Germany, there are no current data available at all [19-21]. Therefore, we intend to close this gap by collecting data in a prospective multicenter study, which should act as a pilot for the planning of a randomized controlled trial (RCT).

Rehabilitation in Germany is a statutorily regulated part of the health care system and is only approved when social participation and inclusion, daily life, and the professional activities of the patient are restricted by illness. Access to inpatient rehabilitation is only possible following a thorough investigation by physicians specializing in social medicine. Proof that all outpatient treatment options have been exhausted must also be provided.

ProKaSaRe (Prospektive Katamnesestudie Sarkoidose in der pneumologischen Rehabilitation) [Prospective Catamnesis Study of Sarcoidosis in Pulmonary Rehabilitation] is a “real-life” cohort trial and a prospective multicentric follow-up (catamnesis) study of sarcoidosis in pneumological rehabilitation. It is designed to assess the impact of a multimodal 3-week inpatient pulmonary rehabilitation on symptoms, exercise capacity, and health-related quality of life (HRQL) for adult patients with chronic sarcoidosis. ProKaSaRe has a 1-year follow-up time.

We intend to rule out seasonal influences by including patients over a recruitment phase of 12 months. By assessing changes over a long period in this cohort of chronic sarcoidosis patients, so far the largest worldwide, we hope to gain reliable insights into and sound evidence of objective and/or subjective short- and long-term health changes caused by pulmonary rehabilitation. The most important questions that should be answered with the ProKaSaRe study are:

1. What are the characteristics of patients treated in inpatient pulmonary rehabilitation?
2. What are the short- and the long-term findings in view of symptoms, exercise capacity, and HRQL?
3. Will the HRQL change after the patients are discharged and how will the HRQL develop in the 12 months following rehabilitation?

The aim of the ProKaSaRe study is to create a solid basis of empirical real-life data on the effectiveness of pulmonary rehabilitation for patients with chronic sarcoidosis, including clinical parameters, exercise capacity, quality of life, and psychological burden via a prospective, multicentric, real-life observational study.

## Methods

### Design

**Figure 1:**
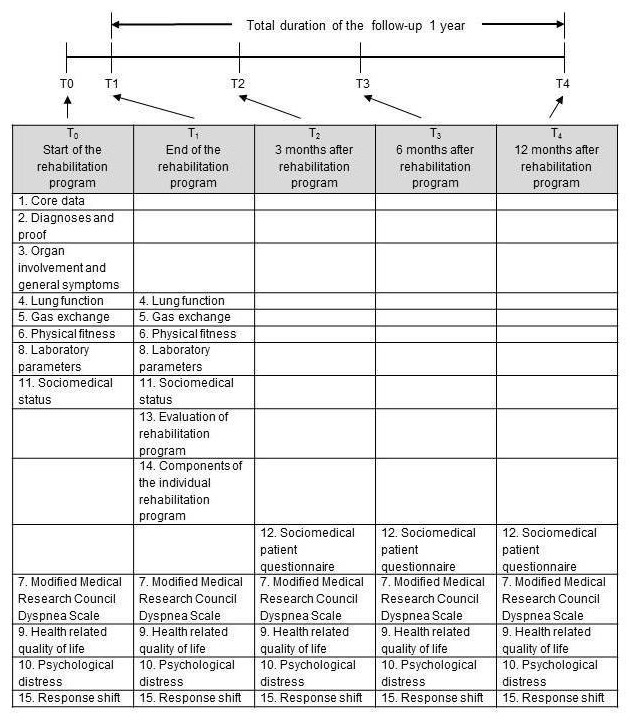
Checkpoints for the different tests, measurements, and distribution of the questionnaire.

ProKaSaRe is a prospective, multicentric observational study. All participants will be observed undergoing the usual structured, multidisciplinary rehabilitation program (Figure 1), which routinely includes a diagnostic assessment consisting of lung function measurements, a 6-minute walk test (6MWT) and various laboratory tests (described subsequently). As part of the study, patients with chronic sarcoidosis will be asked to answer several questionnaires at the beginning and at the end of the usual pulmonary rehabilitation program, as well as after 3, 6, and 12 months.

Ethical approval was granted by the Ethical Committee of the Hannover Medical School, Hannover, Germany (Nr 877).

### Selection of Study Centers and Participants

Rehabilitation clinics specializing in pulmonary rehabilitation of sarcoidosis patients that have already showed interest during a planning conference initiated by the Bad Reichenhall clinic, Bad Reichenhall, Germany, will be formally invited to participate in the trial. All centers have proven sarcoidosis expertise and the selection will be made so that a variety of differing organizational structures, including private clinics and clinics under the auspices of the German statutory pension insurance scheme will be represented.

Based on the referral rate of previous years, we estimate that the participation of 6 pulmonary rehabilitation–specialized centers will be necessary to recruit a sufficient number of patients in 12 months to perform meaningful statistical data analysis.

To be eligible for inclusion in the ProKaSaRe study, patients must be referred for sarcoidosis to one of the 6 participating study centers for inpatient pulmonary rehabilitation by their physicians or by the hospital doctors following acute inpatient hospital treatment. If the leading rehabilitation diagnosis of sarcoidosis is confirmed by a pneumologist expert in sarcoidosis on admission to the rehabilitation program, every patient who is younger than 80 years and aged at least 18 years will be invited to participate in the trial. On receipt of written consent, the patients will be consecutively recruited to and enrolled in the study regardless of their type of health insurance to assess a representative study population.

Patients will only be excluded for poor German language skills, the inability to understand and complete the study questionnaires (due to lack of linguistic proficiency), or if no written consent can be obtained.

### Data Collection and Compilation

Data will be collected at 5 time points: T_0_-T_4_. Defined specific medical measurements and tests will be undertaken at the beginning and the end of the pulmonary rehabilitation program and a questionnaire concerning symptoms and HRQL will be provided at different points in time during the inpatient program and the follow-up period of 12 months (Table 1). Clinical data, sociodemographic data, medication and comorbidities, generic and illness-specific quality of life questionnaires and validated questionnaires documenting anxiety and depression, fatigue, and dyspnea will be monitored on admission at the start (T_0_) and at discharge from the rehabilitation program (T_1_) [22].

At T_0_, the core data will be compiled comprising sociomedical status, age, sex, body mass index (BMI), severity of sarcoidosis, and organ involvement and damage. Previous treatment will also be assessed.

The following clinical findings will be recorded by the doctors at the start (T_0_) and end (T_1_) of the rehabilitation program at each clinic using medical survey forms: lung function, gas exchange, physical fitness, and laboratory parameters. This includes the following measurements:

1. BMI [23]
2. Lung function values: vital capacity (VC), residual volume (RV), total lung capacity (TLC), forced expiratory volume in 1 second (FEV_1_), specific airway resistance (sRaw), mouth occlusion pressure 0.1 seconds after onset of inspiration (P0.1), and maximal inspiratory mouth pressure (Pimax), and Tiffeneau-Index (the FEV_1_/VC ratio) [24]
3. Gas exchange: blood gas analysis at rest and during exercise (25/50 Watt), transfer factor of the lung for carbon monoxide (Tlco) in a single breath (Tlco SB) and per alveolar volume (Tlco/Va)
4. Physical fitness: 6MWT distance [25]
5. Laboratory parameters: angiotensin-converting enzyme (ACE) and C-reactive protein (CRP)

The psychological burden will be also documented at T_0_ using the Hospital Anxiety and Depression Scale (HADS-D) [26] and quality of life by the 36-item Short Form Health Survey (SF-36) and the St George’s Respiratory Questionnaire (SGRQ) [27]. The fatigue symptoms, which are often predominant in sarcoidosis, will be assessed by the Fatigue Assessment Scale (FAS) [28,29] and dyspnea will be measured using the Modified Medical Research Council Dyspnea Scale (mMRC)[30-32]. At 3, 6, and 12 months (T2, T3, and T4) after the rehabilitation program, the FAS, HADS-D, mMRC [30], SF-36, and SGRQ questionnaires will be completed again by the patients. Although the questionnaires at T_0_ and T_1_ will be submitted on site, the ones at T_2_ to T_4_ will be sent by post. The patients will be instructed to complete all questionnaires independently.

In addition, the patients’ views of the components of the individual rehabilitation programs and their perceived importance will be assessed at discharge (T_1_). Moreover, an evaluation of the program from the patients’ perspective will be included in the final questionnaire at T_4._

During the whole study, response shift will be assessed by a questionnaire comprising 5 questions illustrating the prospective hopes of the patient and the retrospective specific burden of disease on the patient.

Data collection by post at 3, 6, and 12 months after discharge from the clinics will proceed by means of standardized letters with enclosed questionnaires and stamped return envelopes. The completed questionnaires will be returned to the clinic at which the rehabilitation program was carried out. Nonresponding patients will be reminded to answer the questionnaire by an additional letter after 2 weeks. Further details and the distribution times of the questionnaires and the documentation of their components are shown in Figure 1 and Table 1.

**Table 1:** Components of the questionnaires given at the beginning of rehabilitation (T_0_), at the end of rehabilitation (T_1_), and at 3, 6, and 12 months after the end of rehabilitation (T_2_, T_3_, and T_4_).

Component	Content
1. Core data	Age, sex
2. Diagnoses	How they were secured or their positive proof
3. Organ involvement and general symptoms	
4. Lung function tests	VC, RV, TLC, FEV_1_, sRaw, Tiffeneau-Index (each before/after bronchospasmolysis), P0.1, and Pimax (T_0_, T_1_)
5. Gas exchange	Resting and exercise blood gas analysis (25/50 Watt), Tlco SB, Tlco/VA, (T_0_, T_1_)
6. 6MWT	6-minute walk test distance
7. mMRC	The Modified Medical Research Council Dyspnea Scale
8. Laboratory parameters	ACE, CRP
9. Health-related quality of life	SGRQ and SF-36
10. Psychological burden	HADS and FAS
11. Sociomedical status	Standardized questionnaire (patients’ self-development)
12. Sociomedical patient questionnaire	Standardized questionnaire (patients’ self-development)
13. Evaluation of rehabilitation program	Evaluation of the rehabilitation program from the perspective of the patient
14. Components of rehabilitation program	Components of the individual rehabilitation program and its (patient) attributed value
15. Response shift	Prospective hopes and the retrospective specific burden of disease

All questionnaires and documents will be anonymized at the 6 participating rehabilitation centers and sent to the Hannover Medical School in Hannover, Germany, for digitalization via independent double data entry by 2 individuals who are not involved in patient care.

### Sample Size Calculation and Statistics

Sample size calculation was carried out separately for both primary endpoints. Because both endpoints are equally important, the type I error was adjusted by Bonferroni multiplicity adjustment.

The minimal clinical difference for the health-related SGRQ corresponds to a change of 4 points in the score for patients with COPD. This value was also used in the planning of this study because no sarcoidosis-specific reference values were available. According to Jones [33], a 4-point difference between baseline values and follow-up data would indicate clinically relevant (long-term) effects. From the 95% confidence interval he observed in 97 patients, we estimated a standard deviation of 12.1. Assuming a 2-sided type I error of 2.5% and a power of 90%, the overall sample size required based on a paired *t* test would be 115 patients.

One estimated minimal clinical difference for the 6MWT distance corresponds to 26 m [34]. In absence of sarcoidosis-specific references, we suggest according to Marcon [35], a 6MWT distance of approximately 600 m (SD 80). Assuming a 2-sided type I error of 2.5% and a power of 90%, the overall sample size on the basis of a paired *t* test is 121. Thus, at least 121 patients are needed to test for both primary endpoints. To avoid a systematic “center bias,” each center should recruit at least 30 patients. Sample size calculations were performed using nQuery Advisor7.0.

### Outcome Measurement

As the primary outcome, the following parameters are defined: (1) HRQL determined using the SGRQ [25,33,36] at T_1_ to T_4_ and (2) exercise capacity / physical ability investigated with the 6MWT at T_1_ and T_2_ [25,37-39]. The choice of the 6MWT rather than the VO_2_max as a measure of physical ability is because of the multicentric real-life study design.

The secondary outcome parameters are: (1) fatigue, the leading symptom of sarcoidosis, investigated using the FAS at T_1_ to T_4_ [28,31,40-43]; (2) the psychological burden assessed with the HADS-D at T_1_ to T_4_ [26]; (3) HRQL assessed by the generic SF-36 health survey questionnaire at T_1_ to T_4_ [27,44-47]; (4) the compiled patients’ views of: the components of the individual rehabilitation programs and their perceived importance at T_1_; and (5) an evaluation of the program from the patients’ perspective at T_1_.

To achieve a 1-year follow-up of patients who return to their homes all over Germany after the end of the inpatient rehabilitation, after T1 validated questionnaires sent by post were chosen as the only data source. Dependent on the outcome of the study, a repeat of the objective measurements (lung function and exercise test) after 3, 6, or 12 months is planned as part of a RCT follow-up study.

### Data Management and Statistical Analysis Procedures

Data management will be carried out by the Centre for Public Healthcare and statistical analysis by the Institute for Biostatistics at the Hannover Medical School.

The questionnaires will be anonymized using a clinic identifier and a consecutive patient number and will be digitized in duplicate at the Hannover Medical School and entered in Microsoft Access. The database tables will then be virtually superposed to identify differences between entries. When discrepancies are found, the data entry will be compared with that of the original questionnaire and corrected so that an accurate master dataset used for the descriptive analysis and the calculation of effect sizes will emerge.

The main analyses will be performed on the total study population. Missing values will be reported and not replaced initially. Changes in both primary endpoints between T_0_ and T_1_ will be evaluated with a paired *t* test and a 2-sided type I error of 2.5%.

Because ProKaSaRe is an exploratory study, for all analyses except for the primary analysis, *P* values will be assessed descriptively and considered to be of importance for *P*<.05. Therefore, no adjustment for multiplicity will be performed. Descriptive evaluation of clinical characteristics will be conducted. Changes from baseline for continuous data will be compared by paired *t* tests and given with 95% confidence intervals for the mean difference. Categorical parameters will be compared for T_1_ to T_4_ by chi-square test. Duration of sick leave and results of the sociomedical status documented by the physician (T_0_, T_1_) and by the patient (T_2_, T_3_, T_4_), the evaluation of rehabilitation from the patients´ perspective (T_1_) and patient-attributed value to the components of the individual rehabilitation program will be presented exploratively. For the questionnaires, a covariate analysis will be run using participating centers and baseline values as independent variables and the change from T_1_ and T_2_ to T_0_ as dependent variables. Further multivariate analyses are planned.

All statistical analyses will be conducted using the SAS 9.3 software (SAS Institute Inc, Cary, NC, USA) to show the impact of the rehabilitation program on the patients’ lives.

#### Data Quality Check

The planned double data entry and the possibility of referring to the clinical files in case of runaway values and spikes concerning lung function, gas exchange, and laboratory parameters should ensure high data quality.

### Intervention

The intervention is an inpatient rehabilitation program lasting 3 weeks. The program is modular, with obligatory and optional components. All components are available at all study centers (Textbox 1). The reasons for choosing these interventions rather than others is described briefly in the following.

Rehabilitation is oriented according to the nature and extent of the difficulties resulting from single (sarcoidosis) or multiple illnesses (comorbidities) that the individual patient suffers that affect professional and daily life. According to these disabilities and functional deficiencies (as defined by the rehabilitation admission tests and assessments), the therapy components are individually and quantitatively adapted.

**Components of the (usual) rehabilitation program carried out by all participating sites in ProKaSaRe.**

Obligatory components

- Specialist confirmation of diagnosis and checks of current medication.
- Endurance training: from 3 to 5 training sessions per week at approximately 60-80% of maximum capacity (walking, power walking, or cycle ergometer).
- Strength training: 3 medical training therapy sessions per week.
- Respiratory physiotherapy: at least three group sessions per week and at least two individual respiratory therapy sessions during the rehabilitation program.
- Patient education: modular sarcoidosis education program.

Optional components

- Inspiratory muscle training: at least five sessions (each 30 minutes) per week, starting at 30% PI_max_. Sessions supervised at least once a week.
- Relaxation treatment: autogenic training or progressive muscle relaxation.
- Social counseling.
- Nutrition counseling.
- Psychological individual and group therapy.
- Structured smoking cessation.
- Ergotherapy and advice on devices and equipment.
- Patient education: additional courses (eg, long-term oxygen therapy training).

#### Obligatory Components

Endurance training is intended to improve performance, shortness of breath, mood, and quality of life. It also compensates for cortisone adverse effects, such as osteoporosis, muscle degeneration, weight gain, diabetes, and high blood pressure [15,48,49], allowing improvement in activities of daily life as defined by the International Classification of Functioning, Disability and Health (ICF) [50]. Cortisone may also affect the mood, sometimes toward depression or irritability.

Respiratory physiotherapy supports the learning of coughing techniques, positions which make breathing easier, handling shortness of breath on exertion, stretching positions to maintain the flexibility of the bony thorax (particularly with fibrosis), correct breathing techniques, and improvements in the respiratory muscles.

Patient education provides the sick person with information on the clinical picture, helps them to cope with their illness, and deal with all the information on adverse effects of their therapies (eg, cortisone). It puts sarcoidosis into perspective and helps to reduce anxiety and depression by generating feelings of informed safety. It explains the reasons for and frequencies of check-ups and helps patients to differentiate between required and unnecessary diagnostic tests. Alternative therapies and their evaluation with respect to sarcoidosis treatment could be addressed. Finally, it generates opportunities for strong interaction and bonding with other patients.

#### Optional Components

Inspiratory muscle training is known to improve the strength of respiratory muscles [51]. Relaxation treatment helps to reduce the symptoms of disquiet, stress, and anxiety.

Social counseling provides advice relating to societal participation, level of disability, severe disability, career reintegration according to social statutes and the ICF, taking early retirement, and applying for benefits, etc [49,52,53].

Nutrition counseling will also be offered on demand, particularly with reference to the adverse effects of cortisone therapy, such as osteoporosis, increased weight, diabetes, and high blood pressure, including counseling on the subject of a healthy diet.

Psychological individual and group therapy will be offered when needed. Individual therapy includes counseling and advice when anxiety, depression, insecurity, and problems coming to terms with the illness occur due to the chronic nature of the illness [54]. Structured smoking cessation will be offered to all smokers. Ergotherapy and advice on devices and equipment will be occasionally appropriate as part of physiotherapy.

## Results

Funding was obtained on October 12, 2010; enrollment began on January 15, 2011 and was completed by January 14, 2012. Results are anticipated late summer 2015.

## Discussion

Sarcoidosis is a systemic disease of unknown etiology that can affect all organs. Therefore, it presents a heterogeneous clinical picture associated with multiple comorbidities. It can occur at any age with a peak between the ages of 20 and 40, with women having a higher frequency of illness than men do, and it can show a chronic or chronic-relapsing pattern. Significant functional limitations include reduced quality of life and an often-debilitating fatigue that hinders the patients’ participation in “normal” daily life. Sarcoidosis is often insufficiently treatable by drugs alone. Can rehabilitation slow down disease progression and accelerate recovery and return to employment? ProKaSaRe seeks to find out whether and to what extent a rehabilitative intervention can substantially contribute to the care of patients with sarcoidosis.

This paper outlines the rationale and design for a prospective, “real-life” multicenter study using obligatory and optional components administered by a multiprofessional team. The aim is to explore the effects of a 3-week inpatient multiprofessional pulmonary rehabilitation program. ProKaSaRe will supply lacking evidence as to the degree to which a training-based multimodal rehabilitation can produce or enhance a significant and sustained improvement to HRQL, physical fitness, and psychological distress. This is particularly relevant in light of the myasthenia and fatigue mentioned previously, which are common symptoms of sarcoidosis.

During 2011, 929 sarcoidosis patients were admitted for inpatient rehabilitation in Germany [55]. We plan to enroll at least 121 consecutive patients within ProKaSaRe, a number that will encompass almost a seventh of all patients undergoing rehabilitation for sarcoidosis in Germany during the year of the study. By using a recruitment time of 12 months, seasonal influences will be excluded, which adds to the validity of the findings.

ProKaSaRe will help to define the patient group currently being referred for pulmonary rehabilitation in Germany, while simultaneously documenting the effect of pulmonary rehabilitation in terms of clinically relevant outcome parameters, if any.

The inpatient rehabilitation that will be assessed consists of a clearly defined set of components developed and approved by experts in the field accredited by the German health system following assessment of their professional development and experience as heads of specialized centers. The detailed agreed-upon choice of components and the broad assessment of HRQL reflecting the subsequent patients’ reaction to their chosen and advised measures represent the strengths of ProKaSaRe.

Scientists and physicians tend to define therapeutic success by only the improvement in pulmonary function, whereas patients equate successful treatment with tangible effects in their daily lives and an improved sense of well-being. This latter ultimately determines long-lasting positive effects, as stated by the ICF [50]. For this reason, both measurements (clinical data and patients’ self-evaluation) are necessary to form a balanced picture of pulmonary rehabilitation outcomes.

Although ProKaSaRe could contain some bias because it assesses only the sarcoidosis population willing to undergo pulmonary rehabilitation and is not a RCT, it is nevertheless a real-life study. Therefore, it is likely that the results generated in ProKaSaRe will be highly generalizable.

In contrast to studies focusing on single nonpharmaceutical components of rehabilitation programs, ProKaSaRe considers the entirety of the pulmonary rehabilitation program. The participants represent a real-life sample of sarcoidosis patients rather than a selected subpopulation thereby adding to the importance of the findings for medical and economical deliberations.

If the results of ProKaSaRe show effectiveness of the rehabilitation over a long period of time, rehabilitation as documented by the study would offer perspective and justified hope to concerned patients and their families and also evidence to stakeholders in the health care system. The documented efficacy of pulmonary rehabilitation for sarcoidosis patients will fill a gap in the reference literature used to develop guidelines and efficient long-lasting treatment procedures. It may establish rehabilitation as an irreplaceable and necessary additional therapeutic option for sarcoidosis, with highly relevant outcomes, which is only effective in its entirety and is more than the sum of single administrated components.

Depending on the outcome of ProKaSaRe, an RCT will be planned to advance knowledge and funding will be applied for. The definition of patient groups to which rehabilitation offers the greatest benefit and the estimation of the financial savings by the prescription of a rehabilitation program would be the next topics to be investigated.

## Abbreviations

6MWT: 6-minute walk test
ACE: angiotensin-converting enzyme
BMI: body mass index
COPD: chronic obstructive pulmonary disease
CRP: C-reactive protein
FAS: Fatigue Assessment Scale
FEV1: forced expiratory volume in 1 second
HADS-D: Hospital Anxiety and Depression Scale
HRQL: health-related quality of life
ICF: International Classification of Functioning, Disability and Health
mMRC: Modified Medical Research Council Dyspnea Scale
Pimax: maximal inspiratory mouth pressure
P0.1: mouth occlusion pressure
RCT: randomized controlled trial
RV: residual volume
SF-36: 36-item Short Form Health Survey
SGRQ: St George’s Respiratory Questionnaire
sRaw: specific airway resistance
TLC: total lung capacity
Tlco: carbon monoxide transfer factor
Tlco SB: carbon monoxide transfer factor in a single breath
Tlco/Va: carbon monoxide transfer factor per alveolar volume
VC: vital capacity
